# Insights into the Fold Organization of TIM Barrel from Interaction Energy Based Structure Networks

**DOI:** 10.1371/journal.pcbi.1002505

**Published:** 2012-05-17

**Authors:** M. S. Vijayabaskar, Saraswathi Vishveshwara

**Affiliations:** Molecular Biophysics Unit, Indian Institute of Science, Bangalore, India; Bar Ilan University, Israel

## Abstract

There are many well-known examples of proteins with low sequence similarity, adopting the same structural fold. This aspect of sequence-structure relationship has been extensively studied both experimentally and theoretically, however with limited success. Most of the studies consider remote homology or “sequence conservation” as the basis for their understanding. Recently “interaction energy” based network formalism (Protein Energy Networks (PENs)) was developed to understand the determinants of protein structures. In this paper we have used these PENs to investigate the common non-covalent interactions and their collective features which stabilize the TIM barrel fold. We have also developed a method of aligning PENs in order to understand the spatial conservation of interactions in the fold. We have identified key common interactions responsible for the conservation of the TIM fold, despite high sequence dissimilarity. For instance, the central beta barrel of the TIM fold is stabilized by long-range high energy electrostatic interactions and low-energy contiguous vdW interactions in certain families. The other interfaces like the helix-sheet or the helix-helix seem to be devoid of any high energy conserved interactions. Conserved interactions in the loop regions around the catalytic site of the TIM fold have also been identified, pointing out their significance in both structural and functional evolution. Based on these investigations, we have developed a novel network based phylogenetic analysis for remote homologues, which can perform better than sequence based phylogeny. Such an analysis is more meaningful from both structural and functional evolutionary perspective. We believe that the information obtained through the “interaction conservation” viewpoint and the subsequently developed method of structure network alignment, can shed new light in the fields of fold organization and *de novo* computational protein design.

## Author Summary

Proteins are polymers of amino-acids that fold into unique three-dimensional structures to perform cellular functions. This structure formation has been shown to depend on the amino-acid sequences. But examples of proteins with diverse sequences retaining a similar structural fold are quite substantial that we can no longer consider such phenomenon as exceptions. Therefore, this non-canonical relationship has been studied extensively mostly by studying the remote sequence similarities between proteins. Here we have attempted to address the above-mentioned problem by analyzing the similarities in the spatial interactions among amino-acids. Since the protein structure is a resultant of different interactions, we have considered the proteins as networks of interacting amino-acids to derive the common interactions within a popular structural fold called the TIM barrel fold. We were able to find common interactions among different families of the TIM fold and generalize the patterns of interactions by which the fold is being maintained despite sequence diversity. The results substantiate our hypothesis that interaction conservation might by a driving factor in fold formation and this new outlook can be used extensively in engineering proteins with better biophysical characteristics.

## Introduction

Proteins are amino–acid polymers capable of folding into unique three–dimensional functional states. The information for the structure formation is contained within their amino–acid sequence [1]. With an enormous amount of data available on genomic sequences in organisms and the structures of the proteins they encode, it has become evident that despite the large sequence space, the structure space is rather limited [2]–[4]. It has been predicted that merely a few thousand protein folds are needed to generate the entire repertoire of the multimillion strong protein universe [5], [6]. The limited number of folds has been explained as a result of optimization of backbone packing [7], [8]. A recent analysis of the fold space showed that the atomic interaction network in the solvent–unexposed core of protein domains are fold–conserved, and that the network is significantly distinguishable across different folds, providing a “signature” of a native fold [9].

As a common rule, homologous sequences generally take up similar folds and the sequence divergences are concomitantly accompanied by structural variations [10]. However, increasing number of identified sequences and folds show a significant departure from this rule, i.e the same fold is able to house highly dissimilar protein sequences [11]–[14]. Folds like the TIM (Triosephosphate Isomerase) barrel, Rossmann, αβ–plait, and all β–immunoglobins are taken up by divergent sequences thereby underscoring the availability of limited fold space. These folds with their simple and symmetric architectures seem to be favorable folds for a large number of non–homologous sequences. Such folds are of special interest since their investigation would provide profound insights into the principles governing protein folding and stability. Although functional variations are related to structural variations, it has been established that proteins with disparate structures may retain their function during the course of their evolution as long as the local active site geometry is maintained [10], [15].

Triosephosphate Isomerase (TIM) Barrel is one the ancient folds with considerable sequence diversity [2]. It is also one of the ubiquitously occurring enzymatic folds and hosts the most diverse enzymatic reactions catalyzing five of the six classes of biochemical reactions [16], [17]. Thus TIM barrel, possessing both structural and functional diversity, has appealed both structural biologists and biochemists equally over the years. Factors responsible for its structural maintenance and functional diversity have been investigated in detail since its first structural discovery in 1975 [16], [18]–[24]. The fold consists of an alternating helix–loop–strand secondary structure motif, where the strands assemble into the core β–barrel. This β–barrel is therefore formed by parallel strands, which is a rarity in fold space [24]. The outer rim of the barrel is maintained by helix–sheet and helix–helix interactions. Evolutionary studies suggest that there are evidences for both divergent [23] and convergent [20] evolution of the TIM barrel proteins, and hence, its evolution is being highly debated. A large number of computational studies have been carried on this fold, focusing mainly on their prevalence in the enzymes of various organisms catalyzing different functions, their structural and evolutionary properties [16], [21]–[26].

In this study we have explored the factors responsible for the stability of TIM fold taken up by dissimilar sequences. Unlike earlier studies that focus on residue conservation, we have focused on interaction conservation as the basis of understanding the underlying structural determinants of the TIM fold. Although this is a novel method, several concepts related to protein sequence-structure-function relationship have been explored and quantitative results have been presented in the literature. For instance, evolutionary concepts were implemented in identifying pair-wise [27] and sets of residues, called as a “sectors”, that have undergone correlated mutations [28] in the protein sequences. At the structure-dynamics level, coarse-grained network models have shown that proteins with similar architecture exhibit similar large-scale dynamic behavior [29] and the differences usually occur in regions where specific functions are localized. Energetic coupling between residues has been investigated both experimentally by mutation followed by biochemical measurements [30] and from computational methods [31]. The classical problem of studying the structure-function relationship in allostery has been addressed from protein structure network point of view [32]–[36]. In essence the protein sequence-structure relationship and the structural changes accommodating their biological function have been investigated by a variety of methods.

Here, we have made the preliminary attempt to study the role of conserved interactions in stabilizing a fold by (a) analyzing residue–residue interactions obtained from atomistic force fields; (b) investigating the interactions and their threshold energy values at a global level by constructing Protein Energy Networks (PEN); (c) obtaining a common PEN for a family of proteins (*f–*PEN) by structure based alignment followed by the construction of a common energy–weighted interaction matrix; (d) using the *f–*PENs to study the conserved interactions responsible maintaining the fold and (e) exploiting the conservation of interactions (obtained from *f–*PENs) to deduce phylogenetic relationship (trees) as opposed to the commonly practiced sequence based methods.

PENs are structure networks where the constituent amino–acids are the nodes and the edges represent the non–covalent interactions among them. By representing the interactions as interaction energies (obtained from molecular mechanics force fields), both the chemistry and the geometry of the amino–acids are better represented than other contact–based structure networks [37], [38]. We have used structural similarities between the remote homologues of TIM barrel fold to align their PENs to obtain information on the extent of interaction conservation among them.

The analysis of *f–*PENs has provided us a wealth of information in terms of the strength of interactions and their conservation (at pair–wise as well as at the level of a collection of multiple interactions). We have been able to identify the factors responsible for the stability of the different secondary structural interfaces in the TIM fold. In general we have observed that the residues involved in high–energy interactions to have more conservation than the residues forming low–energy vdW dominated interactions. We have seen that high–energy conserved interactions are present in the central β–barrel stabilizing it and in the catalytic loop regions helping in the functioning of the protein. The interface between helices and sheets are dominated exclusively by low–energy interactions between non–conserved residues, thus contributing much to the sequence diversity. We also observed that interaction conservation based phylogeny represents the structural and functional evolution better than those derived from sequence conservation. The new outlook from “interaction conservation” has shed more light on the factors behind the fold organization of TIM fold by sequentially diverse homologues. Such observations are unique and we believe that this method will pave an alternate way for understanding the basis of organization of other folds as well. Furthermore, the information on interaction conservation can enable more controlled engineering of new proteins with enhanced structural/functional properties.

## Results/Discussion

### TIM Barrel fold

**Figure 1:**
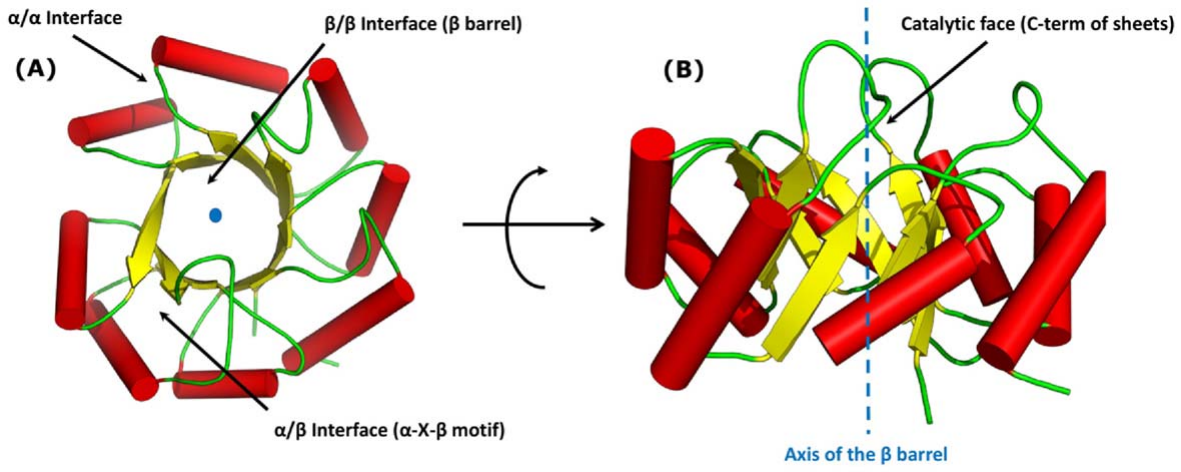
Canonical TIM fold. The canonical TIM fold (αβ_8_) is shown from two different view–points. (A) The three different interfaces namely the β/β encompassing the central β barrel, the α/β and α/α interfaces are highlighted. The face comprising of the C–term end of the β strands, the adjoining loops/turns and the N–term of the helices are broadly classified as the catalytic face of the TIM fold (B), since they feature the catalytic sites.

The TIM fold comprises three major secondary structural interfaces: the central β–barrel, α/β and α/α (Figure 1a). The central β–barrel is formed by staggered parallel β sheets forming the β/β interface and makes up the core of the fold (Figure 1b). The α/β interface flanks the barrel and is formed by the most common α–X–β motif (where X can be any secondary structure like loops and β turns or even separate motifs). The helices interact with each other to form the α/α interface facing the exterior. It has been shown that the face of the fold with the C–terminal ends of the barrel and the adjoining loops contain the active–site residues, thus forming the catalytic face of the fold (Figure 1b) [18]. As mentioned earlier TIM fold is rich in both sequential and functional diversity marking it a viable system for studying sequence–structure–function relationship.

### Protein Energy Networks of the TIM fold

The analysis of the Protein Energy Networks (PENs) provides a rationale to investigate the non–covalent interactions in proteins at various levels such as the interacting pairs (edges), network of connected residues (clusters), nodes connected by a large number of interactions (hubs) as a function of interaction energy. The domains of the TIM barrel fold in the dataset (Table S1) are represented as energy weighted structure networks (PENs), in which the constituent amino–acids are considered as nodes and the edges are weighted based on the non–covalent interaction energies among the amino–acids (Eq 3, Methods Section). Such a representation of PEN, capturing the non–covalent interaction energies at the atomic level, is capable of providing a consolidated view of the forces stabilizing the fold of the protein, yet retaining the details of individual interactions. It is to be noted that highly favorable interactions (for example, −25 kJ/mol) will be referred to as “high–energy” interactions, whereas less favorable interactions (for example, −10 kJ/mol) will be referred to as “low–energy” interactions. A range of unweighted PEN*_e_*s can be generated from the PEN using specific maximum energy cutoffs (*e*) to define the edges (Eq 4, Materials and Methods). It was earlier noted from the PENs of a set of globular proteins that at low energies (e>−10 kJ/mol) the network is dominated by hydrophobic vdW interactions and above this value (e<−10 kJ/mol), the electrostatic interactions starts dominating the edges in the PENs [38]. The ljPENs are generated to focus exclusively on the vdW interactions by excluding the dominant terms of electrostatic interactions. The largest cluster (LC, see Materials and Methods) profiles as a function of *‘e’* for both PENs and ljPENs are provided for the present dataset of 81 TIM barrel domains (Figure S1). It is clear that the domains show three distinct network behaviors as a function of *‘e’* (Figure S1a). In the high–energy region (e<−20 kJ/mol, henceforth denoted as pre–transition region), the LC size is small with the network connected by electrostatic interactions. The size of the LC increases in the intermediate energy region (−20<e<−10 kJ/mol, transition region) following a sigmoidal profile by accruing low–energy vdW interactions and to encampass the whole protein in the low–energy region (e>−10 kJ/mol, post–transition region), where the vdW interactions are dominant, tethering together local pockets of high–energy interactions. The LC profile of ljPENs is similar to PENs except that the mid–transition point is around −7 kJ/mol (Figure S1b), due to the absence of high–energy electrostatic interactions.

### Family specific PENs (*f–*PENs)

**Figure 2:**
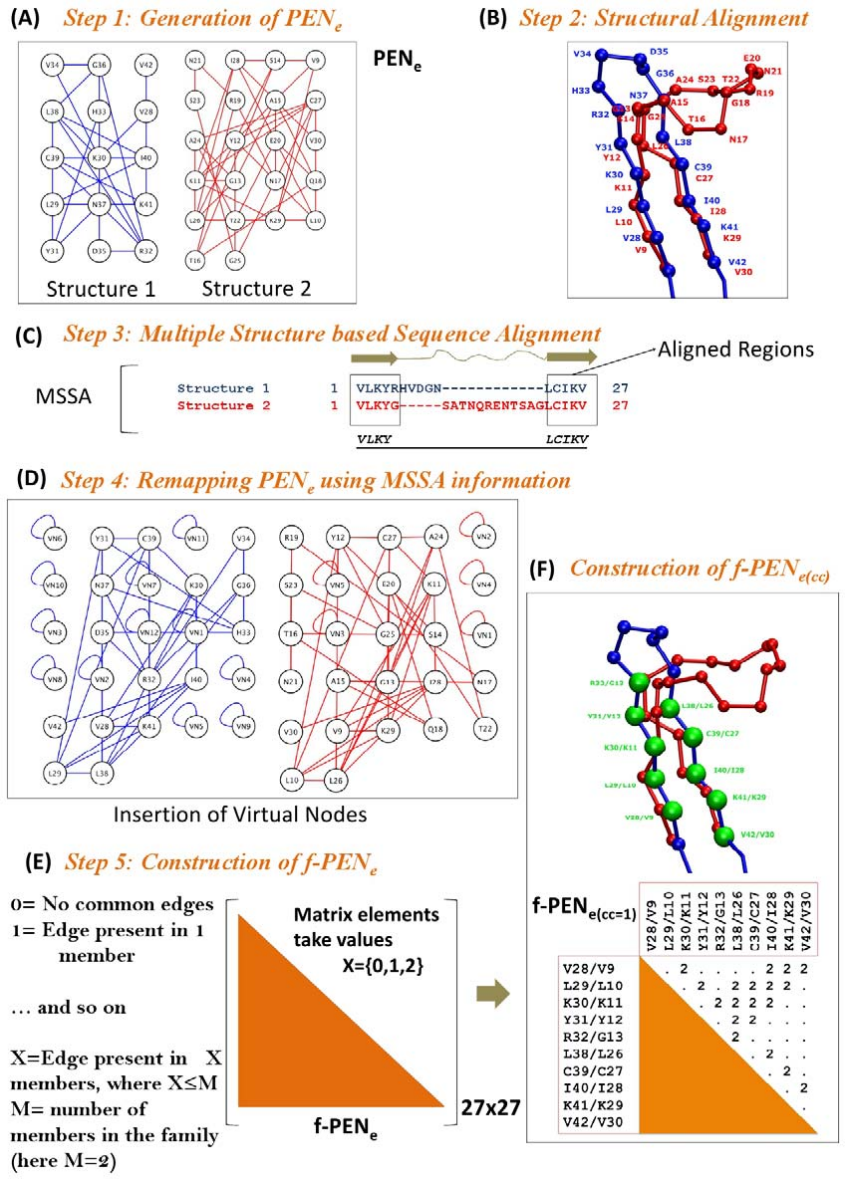
Schematic representation of the construction of family specific PEN at an energy cutoff *‘e’* and commonality coefficient *‘cc’* (*f–*PEN*_e(cc)_*). The steps are indicated with a simple example of two β–loop–β structural motifs, one structure with a short loop (structure 1) and another with a long loop (structure 2). (A) The PEN*_e_*s of the structures (1 and 2) are generated by connecting the residues based on their Cα–Cα distances (however the cutoff energy values (*e*) are chosen in the real cases to draw edges). (B) The structures are superimposed using MUSTANG [51]. (C) The structure based sequence alignment (MSSA) is obtained where the strands are aligned forming a set of equivalent nodes (VLKY and LCIKV) and the non–aligned loops are compensated using gaps in the MSSA. (D) Remapping of PEN*_e_*s of structures 1 and 2 on matrices of the same size (27×27) in which the gaps are represented as virtual nodes (VN, highlighted using self-edges). The arrays of nodes in both the structure networks (red and blue) are equivalent (i.e. Y31 (position 1st row and 2nd column) of structure 1 is structurally equivalent to Y12 of structure 2). (E) The *f–*PEN*_e_* is obtained by aligning both the remapped PEN*_e_* and edges are introduced in the network if they are present in any of the remapped PENs. (F) In this specific case the cc = 1.0 (i e. X = 2), and the family specific network represents only edges that are common to both the structures in the MSSA. The residues involved in the interactions in *f–*PEN*_e(1.0)_* are highlighted as green spheres and the matrix of size 10×10 below the cartoon represents the interactions (X = 2) among the highlighted residues in both the structures.

The TIM barrel domain is a common fold adopted by a large number of diverse sequences. Here we ask the question whether these domains are stabilized by similar patterns of interactions. Despite high sequence diversity we find common patterns of interactions of equivalent energies emerged when investigated at the family level. The family level classification of the TIM fold was obtained from the SCOP database [39]. We constructed family specific PENs for a chosen *‘e’* value (*f–*PEN*_e_*s) (Figure 2) and obtained the equivalent node/edge/network information from the multiple structural alignments of the constituent members (Materials and Methods). Each edge in the family specific network is given a commonality coefficient (*cc_ij_*) value indicating the frequency of occurrence of that edge/interaction in the *f–*PEN*_e_* (Eq 5 and Figure 2f). A ‘*cc*’ value of one corresponds to the presence and a ‘*cc*’ value zero represents the absence of interaction within a spatially similar position of the fold in all the members of a TIM family. Thus various *f–*PEN*_e(cc)_* can be generated for a specific family where *f–*PEN*_e(1.0)_* represents interactions that are present in all the members of the fold and *f–*PEN*_e(0.5)_* represents interactions that are present in at least half the members of the family. In order to determine the role of an amino–acid (node) type in maintaining an interaction (edge), we have used an Entropy based Conservation score (*EC*) for each node in the *f–*PEN (see Methods Section 3.6). Generally if *EC* is greater than zero then there is a degree of conservation of that residue in the family, while a negative *EC* score shows that the residue is not conserved in that position. Therefore, *cc* is a measure of “interaction conservation” between two nodes and *EC* is a measure of “residue conservation” of the nodes.

We have analyzed *f–*PEN*_e_*s in the dataset for edge distribution in different secondary structural interfaces namely the central β–barrel, α/β and α/α interfaces. We further explore the network parameters like clusters and hubs in PENs and *f–*PENs to determine the maintenance of the fold architecture in the TIM fold despite low sequence homology. In our analysis we principally focus on *f–*PENs at the pre–transition region (∼*e*<−18 kJ/mol, Figure S1a) for studying the electrostatic contribution to the fold and the post–transition region of *f–*ljPENs (∼*e*<−8 kJ/mol, Figure S1b) for obtaining the vdW contribution.

### Interactions in the TIM Barrel

By analyzing the distribution of the conserved edges across different interfaces it is possible to determine how the fold is maintained irrespective of the residue conservation.

#### Stabilization of the core β barrel

Apart from the backbone hydrogen bonds, the central β–barrel is stabilized by various other types of interactions like hydrophobic interactions and salt–bridges, arising from the side–chains. Our analyses of f–PENs and f–ljPENs show that there are primarily two modes of the β barrel stabilization. The first mode involves the barrel stabilization primarily due to conserved long–range electrostatic interactions, whereas the second mode involves barrel stabilization due to conserved vdW interactions, the details of which are discussed below.

#### Conserved long range electrostatic stabilization

**Figure 3:**
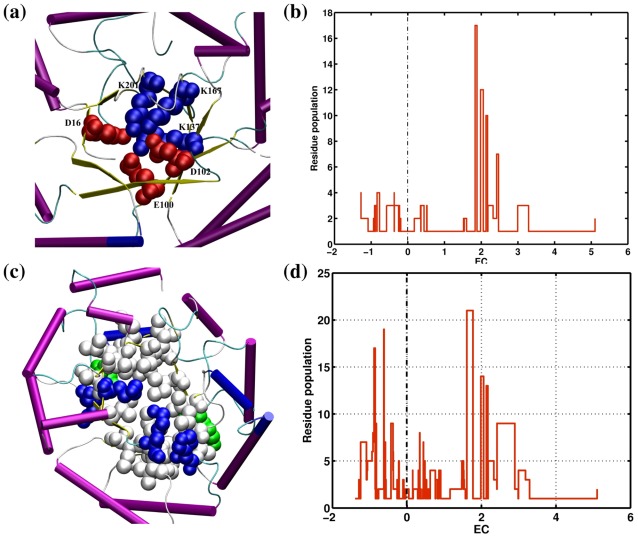
Different modes of stabilization of central β barrel in TIM Barrel families. (a) The cartoon shows the residues involved in the conserved cluster of interactions (*f–*PEN*_–30(1.0)_*) in the C1A family. The residues that are involved in these conserved interactions are highlighted in spheres with blue for basic and red for acidic residues (the representative protein being d1p1xa_). (b) The entropy based conservation indices (*EC*) (obtained from family specific MSSAs (see Methods section)) for residues involved in long range β/β interactions in *f–*PEN*_–15(0.8)_* are given. (c) The cartoon shows the residues involved in low–energy contiguous β barrel interactions formed by the side–chains of residues from adjacent β strands in DC (c.1.2.3) family. The residues that are involved in these conserved interactions are highlighted in spheres with white for hydrophobic and green for polar residues (the representative protein being d1x1za1). Few polar/charged residues (blue) forms vdW interactions with other residues inside the barrel. (d) The *EC*s for the residues involved in contiguous hydrophobic stabilization of the β barrel (obtained from *f–*ljPEN*_–7(1.0)_*) are given. The TIM barrels in (a) and (d) are depicted at an orientation similar to Figure 1a.

In this mode of stabilization, the barrel is maintained by high–energy long–range (sequentially) electrostatic interactions between the side–chains of charged residues. To eliminate the obvious hydrogen–bonded interactions from the neighboring strands, only the interactions between non–neighboring strands are considered as long–range. Figure 3a shows the high–energy (e<−17 kJ/mol), conserved (cc>0.8) long–range β/β interactions that are present in the Class I aldolases (C1A, Table S1). It can be seen from Figure 3a that the charged residues (Asp, Glu and Lys) involved in such conserved long–range electrostatic interactions point towards the center of the barrel. Figure 3b shows the EC scores for the residues involved in such conserved interactions for all families in the dataset. It can be seen that the interactions observed in families like glycosyl hydrolases (FIGH (Table S1)) are maintained by conserved residues within the families as seen from Figure 3b.

#### β barrel stabilization through contiguous hydrophobic interactions

Alternatively the central β–barrel can be stabilized by vdW dominated hydrophobic interactions (observed from f–ljPEN_–8(0.8)_) among the side chains of residues from neighboring strands. Figure 3c shows such interactions from contiguous strands for the Decarboxylases (DC) where hydrophobic residues (Figure 3c) participate significantly in the stabilization of the barrel. Our use of ljPENs for this analysis ensures that the effects of the pre–dominant hydrogen bonds between neighboring strands are masked. The residues involved in such low–energy conserved interactions show both conservation and non–conservation from analysis of their EC scores. Almost all of the families have a certain fraction of very low–energy vdW interactions in the barrel due to the staggered configuration of its parallel strands. However in certain families like Type II Chitinases (c.1.8.5, T2C), Aldo–keto reductases (c.1.7.1, AKC) and Phosphoenolpyruvate mutase/Isocitrate lyase like family (c.1.12.7, PEPM) (Table S1, families 17–19) these conserved interactions solely stabilizes barrel as will be evident in the later sections. It can also be seen from Figure 3a that the aliphatic atoms of the side–chains of charged residues (Lys, Asp and Glu) have packed together contributing to low–energy (vdW) contiguous stabilization of the barrel apart from the charged interactions. Hence the barrel stabilization in C1A family is contributed by both long–range high–energy charged interactions and low–energy contiguous interactions.

#### Stabilization of α/β and α/α interface

The helix–turn/loop–sheet (α/β) is one of the most commonly occurring super–secondary motif in all the α/β folds [2], [39]. We observed that no conserved high–energy α/β or α/α interactions are apparent in most the families, showing that this interface is dominated by low–energy vdW interactions. Figure S2 shows the EC scores for residues involved in these interactions. It was evident from Figure S2 that the residues involved in these conserved α/β interactions are not well conserved across the members of the family unlike the β/β interface (Figure 3a and Figure S2). The reason for such residue non–conservation might be because of the more canonical nature of the α/β motif which can be adopted even by non–homologous sequences across all of the α/β fold class. Therefore we believe that these interfaces might contribute considerably to the sequence diversity of the TIM fold. It has been seen from combinatorial mutagenesis studies on TIM barrels that the central barrel is more sensitive to mutations, whereas mutations at the α/β interfaces are more tolerant, supporting our conclusion that the α/β interface interactions are necessarily not conserved, while β/β interface might require conserved residues to maintain interactions [22], [40].

#### Conserved loop/turn interactions and their functional significance

Within the TIM fold, the loops and turns principally involve themselves catalytically and contribute to its enzymatic diversity [41]. It has long been recognized that the loops of the C–terminal side of the strands have significant number of charged residues and is important for functioning of the TIM fold [22], [40]. Most of the loops that link the C–term ends of the barrel and the N–term of the adjoining helices form the catalytic face of the fold (Figure 1b) and are involved in enzyme catalysis [22], [40]. The loops are structurally more flexible than the helices and sheets, and hence one would expect minimal interaction/residue conservation at the level of loops from structural stability point of view. Interestingly, our analysis showed the presence of high–energy conserved interactions at these regions. We have found that the residues participating in these conserved interactions were predominantly in the catalytic face of the fold in most of the families except AKR (c.1.7.1), T2C (c.1.8.5) and HMGL (c.1.10.5) (Figure S3). This result highlights the importance of conserved interactions in order to maintain the structural features in the flexible loop/turn regions that determine the functionality of the TIM domain. Unlike the high–energy interactions, the low–energy conserved interactions (f–ljPEN_–8(0.8)_) are pervasive; present in both (C–term and the N–term regions of the barrel) the faces of the barrel showing their importance for general structure stabilization.

**Figure 4:**
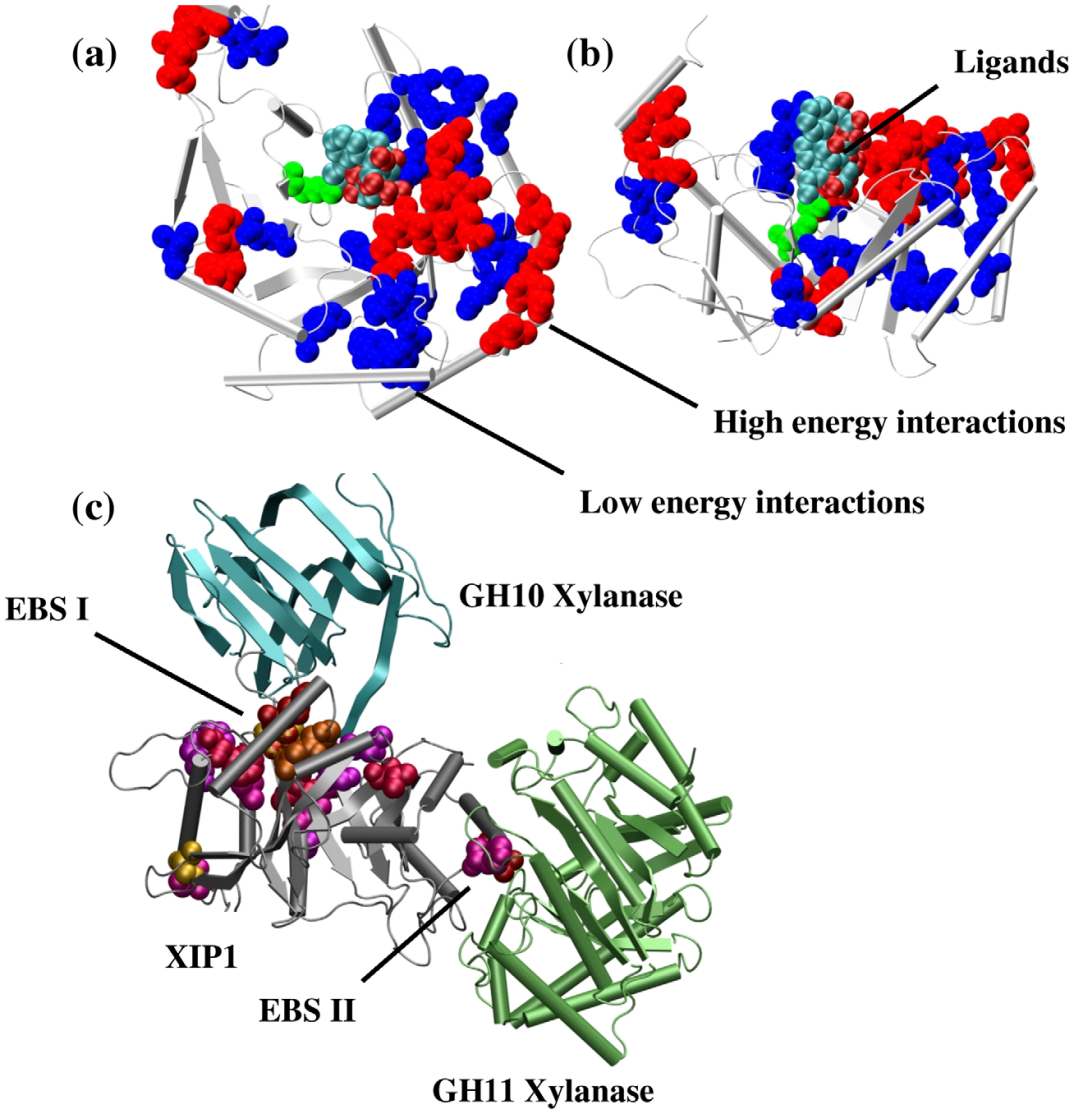
Functional significance of loop based conserved high–energy interactions. The figure depicts the presence of conserved interactions involving loops around the catalytic site in TIM fold. (a) TIM domain from 2mnr was taken as the representative structure for DGDL (c.1.11.2, see Table S1) family. Ligands obtained from close homologues (Protein Data Bank IDs: 1DTN, 1FHV, 1JCT, 1KKR, and 1MDL) were mapped onto 2mnr (after structural alignment of the individual TIM domain and 2mnr) and depicted as vdW spheres colored according to the atom types. The conserved high–energy interactions (*f–*PEN*_–20(0.8)_*) are represented as red spheres while the conserved low–energy interactions (*f–*ljPEN*_–8(0.8)_*) are represented in blue. The important E317 residue which acts as a general acid catalyst in concerted acid–base catalyzed formation of a stabilized enolic tautomer of mandelic acid [56] is highlighted in green. An alternate view of the barrel is given in (b). (c) shows the ternary complex of XIP–GH10–GH11 where the conserved high–energy loop interactions (*f–*PEN*_–20(0.8)_*; the involved residues are highlighted as spheres) in XIP1 (gray cartoon) involved in the inhibitory interactions of GH10 and GH11 (cartoons; cyan and green respectively) at the Enzyme Binding Sites (EBS) are presented.

In order to further investigate the functional significance of the conserved high–energy interactions (*f–*PEN*_–20(0.8)_*) we obtained information on the ligand binding region (catalytic site) by studying the ligand bound structures in the dataset. In the event that ligand bound structures were absent in a family, the ligands were extracted from the structures of closest sequence homologues (>90%) and superimposed onto the representative members in the dataset. Our analysis shows that in families like C1A, PEPM, F1GH, PPL, BNAH, and DGDL (Table S1), conserved high–energy interactions were present in and around the ligand binding site (Figure 4 and Figure S4). For example Figure 4a and Figure 4b shows a representative member (ASTRAL code: d2mnra1) from D–glucarate dehydratase–like family (DGDL, Table S1) with all the ligands (extracted from its nearest sequence homologues) along with the conserved interactions in the loop region. It can be clearly seen that the ligands bind to same site in the catalytic face of the TIM barrel. The conserved interactions both high–energy (*f–*PEN*_–20(0.8)_*) and low–energy (*f–*ljPEN*_–8(0.8)_*) are clustered around the catalytic site, with the high–energy interactions oriented around the charged atoms in the ligands (Figure 4a and Figure 4b). It is evident that these high–energy interactions are conserved so that they can hold the residues in a position to electrostatically interact with the ligands. Figure 4c shows another example where the loop based conserved interactions in a representative member, a Xylanase Inhibitor Protein (XIP1), of the family T2C. It was shown that XIP1 employs substrate mimetic interactions to inhibit two fungal xylanases GH10 and GH11 using two different Enzyme Binding Sites (EBS, Figure 4) [42]. It can be readily seen that conserved loop interactions are present around the EBS of XIP1 probably aiding in the inhibition of GH10 and GH11. It should be noted that we have identified these conserved interactions without the xylanases (GH10 and GH11) in our analysis. Therefore this approach can be helpful in functional annotation of proteins through remote homologues. Figure S4 shows the clustering of high–energy interactions around the catalytic regions in other families.

### Network parameters and *f–*PENs

While the interaction–based studies discussed so far is a step above the residue level investigation, the network parameters like clusters and hubs go beyond pair–wise, by providing a collective view of multiple interacting residues. For instance, even if common interacting pairs in a family of structures are not obvious, a collection of residues interacting at a threshold energy level at similar structural locations can be detected as clusters. Therefore, we have utilized the PENs and *f–*PENs to study certain network properties like hubs and clusters to further understand the formation and stabilization of the fold.

#### Clusters

Clusters in PENs have been shown to represent regions in the protein structures, crucial for stabilization and possibly folding. The clusters calculated at high energies (e<−20 kJ/mol) are compact and electrostatically dominated, while those studied using ljPENs bring out the hydrophobic regions in the protein [38]. Family specific clusters were obtained from the f–PENs (high–energy clusters) and f–ljPENs (low–energy clusters) as described in Materials and Methods Section. From Figure S5 it can be seen that families like C1A (c.1.10.1), DGDL (c.1.11.2), ACD (c.1.8.1), β glycanase (BG, c.1.8.3) and BNAH (c.1.8.6) (Table S1, families 1–5) show groups of high–energy clusters at the central β barrel, whereas families like Decarboxylase (DC, c.1.2.3, Figure 3c), AKR (c.1.7.1), and T2C (c.1.8.5) (Table S1, familes 15, 16, 18) have exclusively low–energy vdW dominated clusters at the barrel devoid of any conserved long–range electrostatic contributions. Families like HPXA (c.1.12.5) and PEPM (c.1.12.7) have both the clusters interspersed throughout the fold (Figure S5). This finding is consistent with the analysis presented on the basis of pair–wise interactions and also shows that these conserved interactions are not isolated but interrelate with each other to form connected sub–graphs. In general, while high–energy clusters form small compact regions predominantly in and around the barrel region and to some extent at the other interfaces like the catalytic face of the fold (Figure S5), the vdW clusters are highly populated and more pervasive throughout the fold.

#### Conserved Hubs

**Figure 5:**
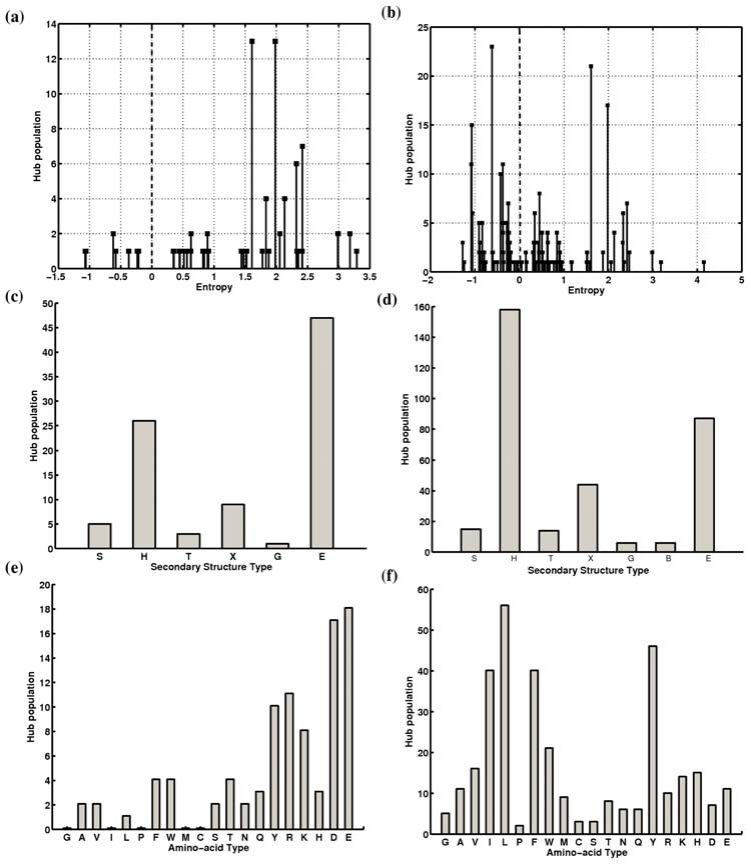
Hub statistics for the different families of the TIM fold. The *EC* scores (a and b), secondary structure type (c and d) and the amino–acid types (e and f) of the conserved hubs identified for the TIM fold families for PEN*_–15(0.7)_* (a, c, and e) and ljPEN*_–7(1.0)_* (b, d, and f).

Hubs which highly connected nodes in a PEN have previously been shown to be crucial for structural stability of proteins [38]. We considered a hub as conserved if the equivalent residues in most of the family members are also hubs. As expected, the total number of conserved hubs is high at lower e and number of hubs decrease as we proceed to higher e. Figure S6 displays the conserved hubs (for PEN_–15_ and ljPEN_–7_) identified in all the members of some of the families. It is generally expected that in globular proteins, the hydrophobic interactions that form the core is highly conserved and the polar residues that are often found in the periphery be less conserved. Interestingly we observed that the charged and polar residues (E, D, R and K) which are predominant in high–energy interactions (Figure 5e), are found to have considerably high EC scores (Figure 5a). The hubs which we obtain from ljPENs, however, behave differently. The amino–acids that occur as hubs are mostly hydrophobic residues (I, L, F, and W) as expected (Figure 5f), with their EC scores suggesting that conservation of residues is not an important factor here (Figure 5b). Only Tyrosine, with the aromatic ring for stacking and a terminal hydroxyl group for maintaining charged/polar interactions, seems to have significant occurrence as conserved hubs in both PENs and ljPENs (Figure 5e and f). Secondary structures that contribute to the hubs mostly come from helices followed by sheets, unlike the hubs in PEN_–15_. This observation suggests that low–energy conserved hubs are at the helix–sheet or the helix–helix interface for maintaining the stability of the interactions around the barrel, whereas conserved hubs capable of high–energy interactions are present at the barrel itself (Figure 5c and d). This observation is highlighted in Figure S6 which shows the structural positions of different hubs in some of the families of the TIM fold. It is evident that the electrostatically dominant hubs (from PEN_–20_) are prominent within the central β–barrel (see families DGDL, ACD) while some families like HMGL show that the vdW dominated hubs (from ljPENs) dominate the barrel interface. Certainly the population of the vdW hubs is very high at the α/β and α/α interfaces.

### Interaction network based phylogeny

**Figure 6:**
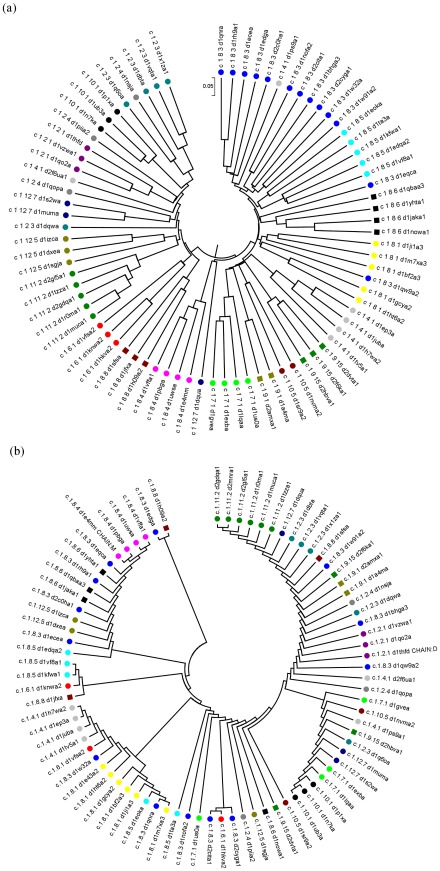
Comparison of network and sequence based cladograms. A comparison of interaction based and sequence based phylogenetic analysis. (a) The cladogram of the hierarchical clustering of the members from network similarity scores (Methods Section). (b) The cladogram of the sequence based phylogeny. For sequence based phylogeny a Maximum Likelihood based statistical method was used for phylogenetic reconstruction.

One of the major implications in understanding protein sequence–structure–function relationship is that we can obtain a variety of evolutionary information. Classically, existing phylogenetic methods exploit sequence conservation information to infer relationships and recent increase in structural data has resulted in the inclusion of structural features to deduce relationships between proteins [43]. The most commonly used sequence conservation based methods fail to obtain correct relationships between remote homologues due to the misgivings of sequence alignment techniques in the “twilight region” of the sequence–structure space. Here we deduce improved similarity relationships between remote homologues of the TIM fold through quantification of the similarity of interactions (edges) from their PENs (details described in Materials and Methods). Figure 6 shows the comparison of the cladograms (a map of the hierarchical clusters) obtained from the interaction based and sequence based techniques. It can be readily seen that the interaction conservation based method clusters proteins of the same family under the same clade better than the sequence conservation based method. It should be noted that the SCOP classification of families is based on sequence or structure or functional similarities. The interaction based phylogeny matches very well with the SCOP classification than the sequence based method for the same dataset. Despite low sequence identity (≤30%) we were able to find domains that exhibited as high as ∼85% interaction conservation (between d1r0ma1 and d1muca1 from DGDL family). These observations show that the interaction based phylogenetic tree may be able to cluster the members of the family better than a residue based classification scheme.

**Figure 7:**
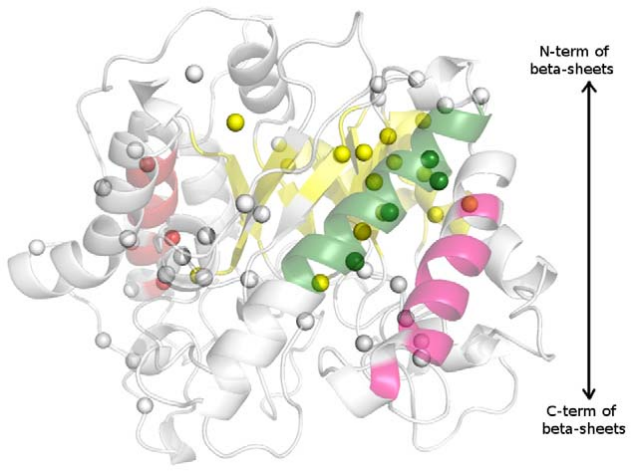
Comparison of *f*–PEN clusters and SCA sectors for β–glycanase family. A comparison of the cluster residues obtained from *f*–PEN and the Sector residues identified from SCA of β–glycanase family. The residues are mapped on the backbone cartoon structure of bacterial cellulose catalytic domain (PDB id: 1EDG). The top four clusters are rendered in yellow, green, magenta and red color backbone representation. The yellow, green, magenta and red spheres are the Sector residues from SCA matching with residues in the clusters of corresponding color. The grey spheres are from Sector, which do not match with the cluster residues (similarly, there are cluster residues which do not match with the Sector residues).

Lockless and Rangathan [27] introduced a sequence-based method to investigate statistical interactions between residues (Statistical Coupling Analysis (SCA)). Later Halabi *et al.*, grouped these statistically correlated amino-acids into quasi-independent groups called sectors and studied their characteristics in Serine proteases [28]. Here we have made the preliminary attempt to compare the interaction-energy based approach with the sequence based SCA approach. We selected β-glycanase family of TIM fold for this comparison. The interactions (≤−10 kJ/mol) common to this family were identified and cross verified with correlated mutations obtained from SCA. Although the correlation appeared to be weak at the pair-wise level, significant correlations are identified when the collective behavior of these correlated pairs are examined. In other words, there is a significant match between the residues of the sector from SCA and the clusters obtained from the present energy based analysis. The results have been pictorially depicted in Figure 7 (details of the underlying calculations and comparison are provided in Table S2 and Table S3). Interestingly, the agreement is more in the regions stabilizing the structure. The residues located more towards the function are identified by SCA and the PEN clusters encompass more of the residues required for the structural integrity. Based on this reasonable correlation of the SCA sectors and PEN clusters, we emphasize the fact that the protein structures should be viewed as a collective entity and an examination of individual residues and pair interactions in isolation may not always provide a holistic view of the structure and function of proteins. This feature was also reiterated by the coarse-grained network model studies on Rossmann-like domain proteins [29]. A weak agreement of pair-wise correlations from SCA predictions with the biochemical experiments on double mutants of PDZ domain perhaps may be attributed to this reason. Furthermore, fundamental issues like divergent [23] or convergent [20] evolution of proteins like TIM barrel, whose sequences are so diverse, has always been debated [16]. Extensive investigation by complimentary approaches such as PEN, SCA and essential mode dynamics should be able to provide more clarity into such systems.

### Conclusions

The sequence–structure relationship is a well–researched area, however, the factors that drive highly diverse sequences to fold into the same structure has not been well understood because of the apparent absence of consensus information from sequence similarity analyses. Here we have taken an alternative approach in which we consider “interaction conservation” and analyze whether the preservation of interactions is an essential driving force in the formation of the fold rather than sequence conservation. TIM barrel fold is one of the most popular folds that have a high sequence variability and functional diversity. In this study we have analyzed non–homologous members of different families of the TIM fold and investigated various factors that contribute to the formation of the fold.

We have adapted the concept of interaction networks in order to study these protein structures from a global perspective. Also, by using interaction energies we have realistically represented the residue–residue relationships in the network. The subsequent methodology that exploits structural alignment to align the Protein Energy Networks (PENs) in a family of TIM fold has provided us with valuable information on the conservation of interactions in the family.

It was evident from our analyses of conserved interactions that the central β barrel is being stabilized by (a) sequentially long–range conserved high–energy interactions and (b) low–energy vdW interactions from residues of the neighboring strands interacting in tandem, in addition to the hydrogen–bonding network in the sheet. Also, the analysis of the other interfaces like the α/β and the α/α show an absence of any high–energy conserved interactions, and being maintained exclusively by low–energy interactions. In general we found that the residues involved in high–energy interactions are better conserved than low–energy interactions. From our cluster analysis it was seen that the conserved interactions are not segregated into isolated interacting pairs but rather coalesce together to form a sub–network of interactions. Our hub analysis has shown that the charged and the conserved residues are favorable to be hubs at higher energies, while hydrophobic residues with less conservation act as hubs at lower energies. All these results suggest that (a) the β barrel formation driven by high–energy interactions (with the participating residues being conserved) seem to be an important step in the organization of the TIM barrel; (b) the formation of the other interfaces mainly by low–energy interactions (with residue conservation being immaterial) is a more canonical step in the fold formation common to all the folds of the α/β class, and can be taken up by a variety of sequences, thus contributing the high sequence diversity. These conclusions concur with several experimental observations that suggest that while the α/β interfaces in TIM are resilient to mutations the β barrel is sensitive [18], [40], [41], [44].

We have analyzed the structural and functional relevance of conserved interactions in the regions involving loops in various TIM barrel families. We found that loop based high–energy conserved interactions (e<−20 kJ/mol) are present near the active sites of a number of TIM barrel families. This suggests that the loop based interactions are conserved during evolution to maintain the active site geometry for successful enzymatic functioning of the TIM proteins. Therefore this method can be used in functional annotation of hypothetical proteins in cases where there are structural homologues but no sequence homologues. Finally we exploited the concept of “interaction conservation” to construct a cladogram and compare it with the sequence based cladogram. The outcome of analysis reinforces our assumption that it may be interaction conservation and not necessarily sequence conservation that determines the fold organization. Our attempt to correlate our method with that of SCA suggests that there may be significant correlation between the sector residues and cluster residues. However, extensive investigation by complimentary approaches such as PEN, SCA and Elastic Network Models (ENM) should be carried out and such an analysis will be able to provide more clarity to studying such protein systems.

The methodology of representing the protein structures as interaction energy based networks and using structural alignments to align these networks has provided us a very convenient handle to study structure homology among sequentially diverse proteins, from a network point of view. We were able to study the salient features that stabilize the TIM fold using this method, and also analyze how interaction conservation can play an important role in the formation of this fold. We believe that this methodology can shed valuable knowledge on the fold maintenance by remote homologues and pave way for useful *de novo* design and analysis of protein folds.

## Materials and Methods

### Dataset

The dataset used in this analysis is composed of domains from the TIM fold given by Structural Classification Of Proteins (SCOP) [39]. The coordinates for the domains are obtained from ASTRAL [45]. The domains are sorted into their respective families as given in SCOP. The sequence identity within the members of each family is less than 30%. The culling of domains with higher sequence identity was done using *cd–hit* [46]. All the families constitute at least three members (except HMGL like domains (HMGL) and Adenosine/AMP deaminase (ADA) families, (see Table S1)). The dataset consisting of 19 families with 81 domains is presented in Table S1. The secondary structural elements (SSE) for each domain were assigned using DSSP [47].

### Methods of generating interaction matrix

Structure network construction requires the coordinates of the interacting amino acids (nodes) and a criterion to define the interactions (edges). A purely geometry based all-atom interaction can be deduced from the crystal structure, which we had used to describe the Protein Structure Networks (PSNs) [48]. Recently, we have considered the chemistry in greater detail by explicitly considering the interaction energy between residues [38]. Although qualitative results are expected to be similar from both formalisms, PEN has the advantage of capturing subtle details of importance, whereas the PSN approach has the advantage of being simple to adopt (Figure S7). The interaction energies can be obtained on a single structure or on an ensemble of structures of a given protein. The set of structures can be obtained from experiments (X-ray crystallography, Nuclear Magnetic Resonance) under different environment or by simulations from a single starting conformation. In the case where the conformational changes are small, a set of conformations will provide a statistically relevant average structure and in the case of large conformational change, it is advantageous to study them independently to characterize the structural variations in different states of the same protein, for example to understand the effect of ligand binding. In this study we have used Molecular Dynamics (MD) simulations to obtain the structure ensemble for each of the TIM domains.

### Molecular Dynamics (MD) simulations

We have considered the crystal structures for all the proteins in the dataset (Table S1) and subjected them to minimization and Molecular Dynamics simulations for a brief time interval (20 *ps*) to obtain interaction energies in equilibrium. In our earlier studies we have shown that the correlation between interaction energies calculated using the equilibrated structures from 2 *ns* simulations and 20 *ps* simulations was around 90% [38]. The MD simulations were performed using GROMACS (GROningen MAChine for Simulations) [49] for just 20 *ps* and structure ensemble for each domain is obtained by sampling its trajectory every 1 *ps*. The average interaction energies among the amino–acids are computed using the structure ensemble thus obtained. Selenomethionines (MSE) present in certain domains like d1pbga_ and d1uwsa_ from Glycosyl hydrolase family (F1GH) were converted to Methionine and missing atoms in the residues were generated using Swiss PDB viewer [50]. The best conformations for both the modified and the built residues recommended by the Swiss PDB viewer from its rotamer library were used.

### Protein Energy Networks (PENs)

The details of the construction of PEN are given in Vijayabaskar and Vishveshwara [38]. Briefly, the non–bonded interaction energies (*E_ij_*, Eq 1) between all pairs of residues were obtained as a summation of the electrostatic (given by columbic potential, Eq 2) and van der Waals (given by the Lennard Jones (LJ) potential, Eq 3) interaction energies averaged over the structure ensemble. PEN is constructed with amino–acids as nodes, and with edges drawn between all pairs of residues except the sequential neighbors. The edges are weighted with the calculated *E_ij_*. ljPENs take into account only the van der Waals (vdW) interactions (i.e *E_ij_ = V_LJ_*). Unweighted networks (PEN*_e_* and ljPEN*_e_*) can be obtained for a specific maximum energy cutoff *‘e’* as given in Eq 4. 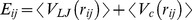
(1)

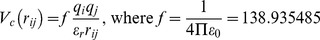
(2)

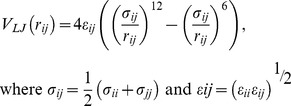
(3)

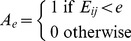
(4)


### Network alignment and family specific energy networks (*f–*PENs)

Steps involved in the construction of the family specific PEN (*f–*PEN) by alignment of the PEN*_e_*s of its members is given in detail in Figure 2. Domains in a family are structurally aligned using MUSTANG (MUltiple STructural AligNment AlGorithm) [51] (Figure 2b). A family specific Multiple Structure based Sequence Alignment (MSSA) was obtained for all the members of a given family and the residues that are aligned in the MSSA are referred to as Equivalent residues. Residues that were not structurally super–imposable were compensated within the alignment using gaps (Figure 2c). The PEN*_e_*s are remapped using the equivalent node information obtained from the MSSA (Figure 2d). The gaps in the MSSA are introduced as virtual nodes in the corresponding PEN*_e_*s, such that the edge weights of a virtual node to all other nodes in the PEN were highly unfavorable (E*_ij_* = 100 kJ/mol where either *i* or *j* is a virtual node) (Figure 2d). The remapped PEN*_e_*s are then aligned to form the family specific PEN (*f–*PEN*_e_*) (Figure 2e) such that the nodes are equivalent and edges exists only if they were present in any of the realigned PEN*_e_*s (Figure 2f).

### Commonality Coefficient (*cc*)

In a *f–*PEN*_e_*, the values (X, Eq 5) of the edges can vary from 0 to *M*, where 0 represents the absence of an edge in all the members of the *f–*PEN*_e_* and *M* represents the edge being present in all members. Therefore each edge is given a commonality coefficient (*cc_ij_*, Eq 5), and it represents the measure of the frequency of occurrence of an edge between equivalent nodes within the members of a family.
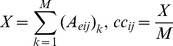
(5)where *X* is the total number of members having the edge between nodes *‘i’* and *‘j’* with interaction energy better than *‘e’*, *Ae_ij_* is the element of the adjacency matrix of the remapped PEN*_e_* and *M* is the total number of members in the family (Figure 2e).

Thus, a family specific PEN can be denoted as *f–*PEN*_e(cc)_* where *‘e’* is the interaction energy cutoff used to generate PEN*_e_*s for all the members of the family and edges are constructed only if their *cc_ij_* is better than ‘*cc*’. The *f–*PEN*_e(cc)_* consists of both equivalent and virtual nodes and represents spatially conserved interactions across the members of that family. In fact both the *‘e’* and ‘*cc*’ values can be used as weights in order to construct a weighted matrix. However, in this study, we have considered un-weighted matrix at given values of *‘e’* and ‘*cc*’.

### Entropy based Conservation Index (EC)

Entropy based Conservation scores (EC) for each alignment position in the MSSA were obtained using AL2CO [52]. In this method the entropy is normalized with the mean and standard deviation. Thus better the entropy score, the more conserved the amino–acids are at that position.

### Interaction network based phylogeny

A network similarity matrix (*S*) for any two members *‘a’* and *‘b’* in the dataset is constructed as given in Eq 6. *S* is an adjacency matrix which takes a value of 1 if the interaction energies between equivalent residues in the MSSA are similar. The Similarity Score (*SS_ab_*) between the PENs of any two members in the dataset is derived as given in Eq 7. This value is the fraction of edges that is conserved between the two members. The distance matrix (*D*, Eq 8) with each row and column representing a domain in the dataset, is used to construct the phylogenetic tree.
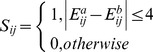
(6)

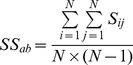
(7)

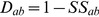
(8)where *E^a^* and *E^b^* are PENs of any two members in the dataset that are remapped based on their pairwise MSSA, and *N* is the total number of nodes in the remapped PENs.

The concept of structure conservation is often used in structural alignment methods [53], [54]. For instance, an alignment based on dynamic characteristics of structurally similar but functionally distinct proteins have been reported earlier [29]. The identification of energetically similar edges in two proteins done in the present study, can also serve as a basis for alternate method of structural alignment, although it is not pursued in this study.

### Clusters and Hubs

Clusters were generated using Depth First Search (DFS) algorithm [55]. Family specific clusters in a family of TIM fold are connected sub–graphs present in the *f–*PEN*_e(cc)_* with a size of at least three (i.e. isolated pair–wise interactions are not considered as clusters). The Largest Cluster (LC) in a PEN*_e_* is the cluster with highest number of constituent nodes. Degree which is the total number of edges incident on a node, is a measure of connectivity of that node in the network. Hubs are defined as nodes with higher degree. The family specific hubs are those residues which are spatially equivalent and have a degree of at least 3.

## Supporting Information

Figure S1 **Largest Cluster (LC) transition profile for PENs and ljPENs of the TIM barrel domains.** The transition of the Largest Cluster (LC) as a function of energy cutoff *‘e’* for PENs (A) and ljPENs (B) of the domains of the TIM fold is given. The LC sizes are normalized with the protein size and the averages are plotted. The error bar indicates the standard deviation of the sizes from their mean values. (A) The figure shows the pre–transition (red), transition and post–transition regions (blue) in the PENs of the domains.
(PDF)

Figure S2 **Conserved α/β and α/α interactions in different families of the TIM fold.** (A) The bar diagram shows the *EC* of the residues involved in conserved interactions that participate in HE interactions in different families of the TIM barrel. Their distribution shows that the conservation interacting residues are very well dispersed. The distribution of the *EC* scores for the residues involved in conserved HH interaction in the *f–*PEN*_–4(0.8)_* (B) and *f–*PEN*_–10(0.8)_* (C) for different families of the TIM fold are shown in the inset figures. The residues seem to be non–conserved across the members of the families.
(PDF)

Figure S3 **Presence of conserved high–energy interactions at the catalytic face of the TIM fold.** The residues participating in the conserved interactions (*f–*PEN*_–20(0.8)_*) at the loop regions of different families of the TIM fold are highlighted in various shades of red.
(PDF)

Figure S4 **Role of conserved interaction in loops in the catalysis of TIM fold.** The above figure shows the conserved high–energy interactions involving loops (*f–*PEN*_–20(0.8)_*) in different families. The ligands are represented in vdW spheres colored according to their atom types while the residues involved in the conserved interactions are highlighted in different shades of red.
(PDF)

Figure S5 **Family specific clusters from the** ***f–*** **PENs for selected families of the TIM fold.** Clusters obtained from *f–*PENs are highlighted as spheres in different families of the TIM fold. High–energy clusters involving charged interactions at the core of the core β barrel residues that are obtained from the *f–*PENs*_–25(0.8)_* are distinguished by different shades of red. Low–energy vdW clusters that are obtained from *f–*ljPEN*_–8(1.0)_* are highlighted in different shades of blue in different families of the TIM fold.
(PDF)

Figure S6 **Conserved hubs present in some of the families of the TIM Barrel.** The above figure shows the conserved hubs present in D–glucarate dehydratase–like (c.1.11.2, DGDL), Amylase (c.1.8.1, ACD), Aldo–keto reductases (c.1.7.1, AKR), HMGL–like (c.1.10.5, HMGL) and beta–N–acetylhexosaminidase (c.1.8.1, BNAH) families of the TIM Barrel domain. The hubs that are in shades of blue are from *f–*PEN*_–15(0.7)_* and that highlighted in shades of red are from *f–*PEN*_–7(1.0)_*.
(PDF)

Figure S7 **Comparison between Cα distances between residues and the corresponding interaction energies.** The above scatterplot shows the distances between the Cα atoms of residues plotted as a function of their interaction energies. It can be seen that although the interaction energy decreases as the cartesian distances between the Cα atoms decreases (green arrow), a number residue pairs fail to follow this behavior (red arrow, blue arrow and the points above the green arrow). This behavior can be attributed to high energy electrostatic interactions, cation-pi interactions, pi-pi interactions etc. that behave differently from contact based vdW interactions. Therefore, although most of the topology-based networks behave very well in studying many biophysical characteristics of proteins, we believe that PENs are capable of capturing the variations in the protein structures brought about by non-vdW interactions. In other words, while contact based networks are good at representing the width of the well that describes the interactions among protein residues, the energy based networks are capable of representing the depth of the well. (It should be noted that any Cα- Cα distance greater than 20 Å is considered as 20 Å and any *E_ij_* less than −30 kJ/mol is considered as −30 kJ/mol).
(PDF)

Table S1 **Different families of the TIM fold taken for analysis.**
(PDF)

Table S2 **Dataset taken for SCA calculation.**
(PDF)

Table S3 **Comparison of the “cluster residues” from** ***f*** **-PEN and the “sector residues” from SCA in β-glycanase family.**
(PDF)
