# Supplementary material for: Insights into the Fold Organization of TIM Barrel from Interaction Energy Based Structure Networks
Source: PLoS Comput Biol. 2012 May 17;8(5):e1002505. doi: 10.1371/journal.pcbi.1002505 (PMC3355060; doi:10.1371/journal.pcbi.1002505)
Supplement: Figure S1 — Largest Cluster (LC) transition profile for PENs and ljPENs of the TIM barrel domains. The transition of the Largest Cluster (LC) as a function of energy cutoff ‘e’ for PENs (A) and ljPENs (B) of the domains of the TIM fold is given. The LC sizes are normalized with the protein size and the averages are plotted. The error bar indicates the standard deviation of the sizes from their mean values. (A) The figure shows the pre–transition (red), transition and post–transition regions (blue) in the PENs of the domains. (PDF) [file pcbi.1002505.s001.pdf]

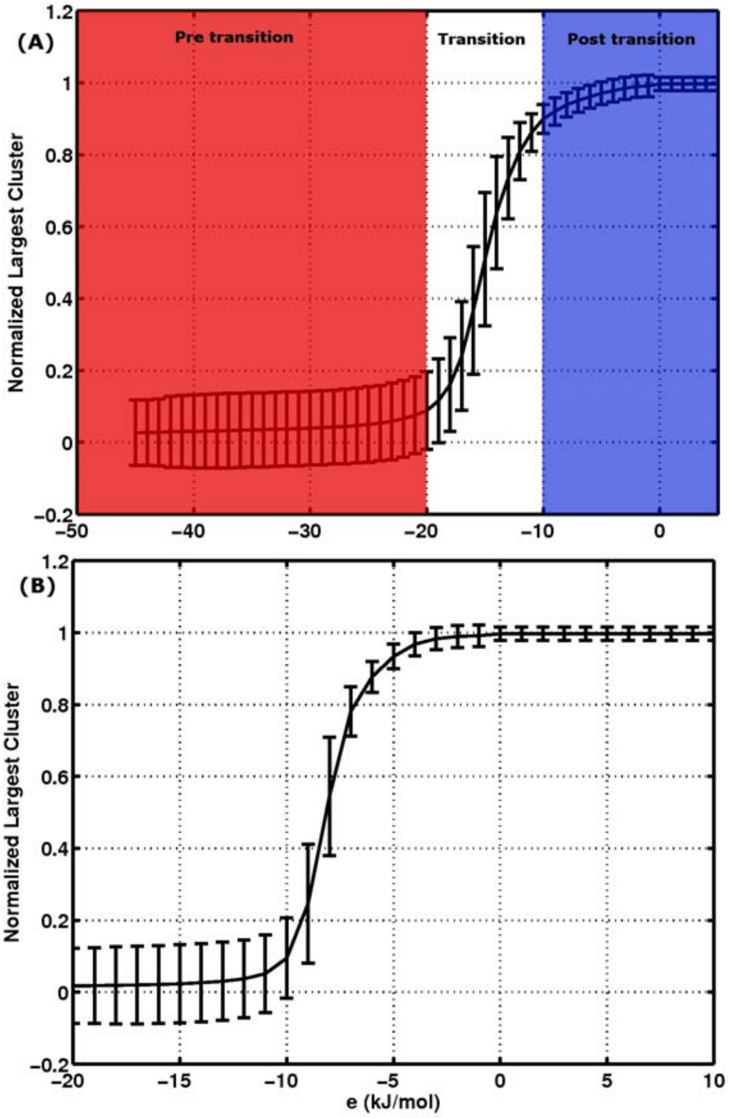

Figure S1 - Largest Cluster (LC) transition profile for PENs and ljPENs of the TIM barrel domains

The Largest Cluster (LC) transition profile as a function of energy cutoff ‘*e*’ for PENs (A) and ljPENs (B) of the domains of the TIM fold is given. The LC sizes are normalized with the protein size and the averages are plotted. The error bar indicates the standard deviation of the sizes from their mean values. (A) The figure shows the pre-transition (red), transition and post-transition regions (blue) in the PENs of the domains.
