# Supplementary material for: Insights into the Fold Organization of TIM Barrel from Interaction Energy Based Structure Networks
Source: PLoS Comput Biol. 2012 May 17;8(5):e1002505. doi: 10.1371/journal.pcbi.1002505 (PMC3355060; doi:10.1371/journal.pcbi.1002505)
Supplement: Figure S2 — Conserved α/β and α/α interactions in different families of the TIM fold. (A) The bar diagram shows the EC of the residues involved in conserved interactions that participate in HE interactions in different families of the TIM barrel. Their distribution shows that the conservation interacting residues are very well dispersed. The distribution of the EC scores for the residues involved in conserved HH interaction in the f–PEN–4(0.8) (B) and f–PEN–10(0.8) (C) for different families of the TIM fold are shown in the inset figures. The residues seem to be non–conserved across the members of the families. (PDF) [file pcbi.1002505.s002.pdf]

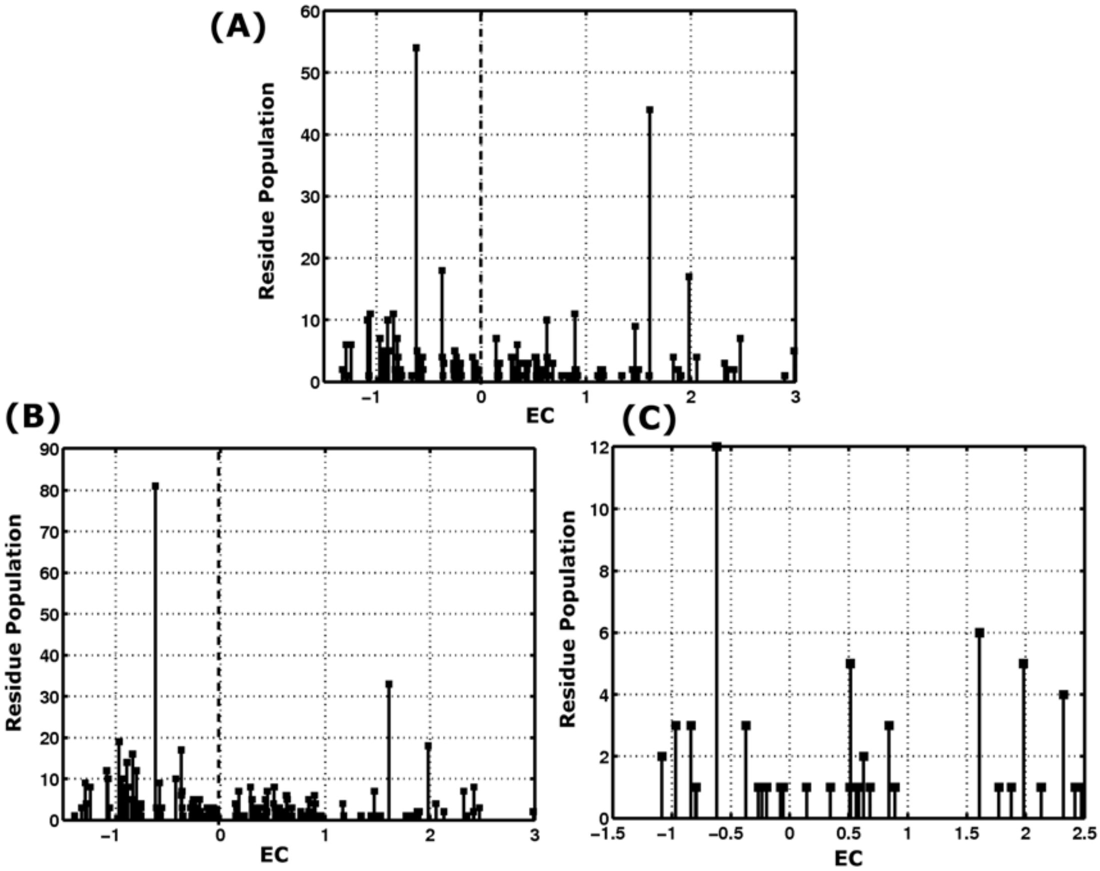

Figure S2 Conserved α/β and α/α interactions in different families of the TIM fold.

(A) The bar diagram shows the EC of the residues involved in conserved interactions present at the α/β interface in different families of the TIM barrel fold. The distribution of the EC scores for the residues involved in conserved α/ α interactions in the *f*-$PEN_{-4(0.8)}$ (B) and *f*-$PEN_{-10(0.8)}$ (C) for different families of the TIM fold are shown. The residues seem to be non-conserved across the members of the families.
