# Supplementary material for: Insights into the Fold Organization of TIM Barrel from Interaction Energy Based Structure Networks
Source: PLoS Comput Biol. 2012 May 17;8(5):e1002505. doi: 10.1371/journal.pcbi.1002505 (PMC3355060; doi:10.1371/journal.pcbi.1002505)
Supplement: Figure S3 — Presence of conserved high–energy interactions at the catalytic face of the TIM fold. The residues participating in the conserved interactions (f–PEN–20(0.8)) at the loop regions of different families of the TIM fold are highlighted in various shades of red. (PDF) [file pcbi.1002505.s003.pdf]

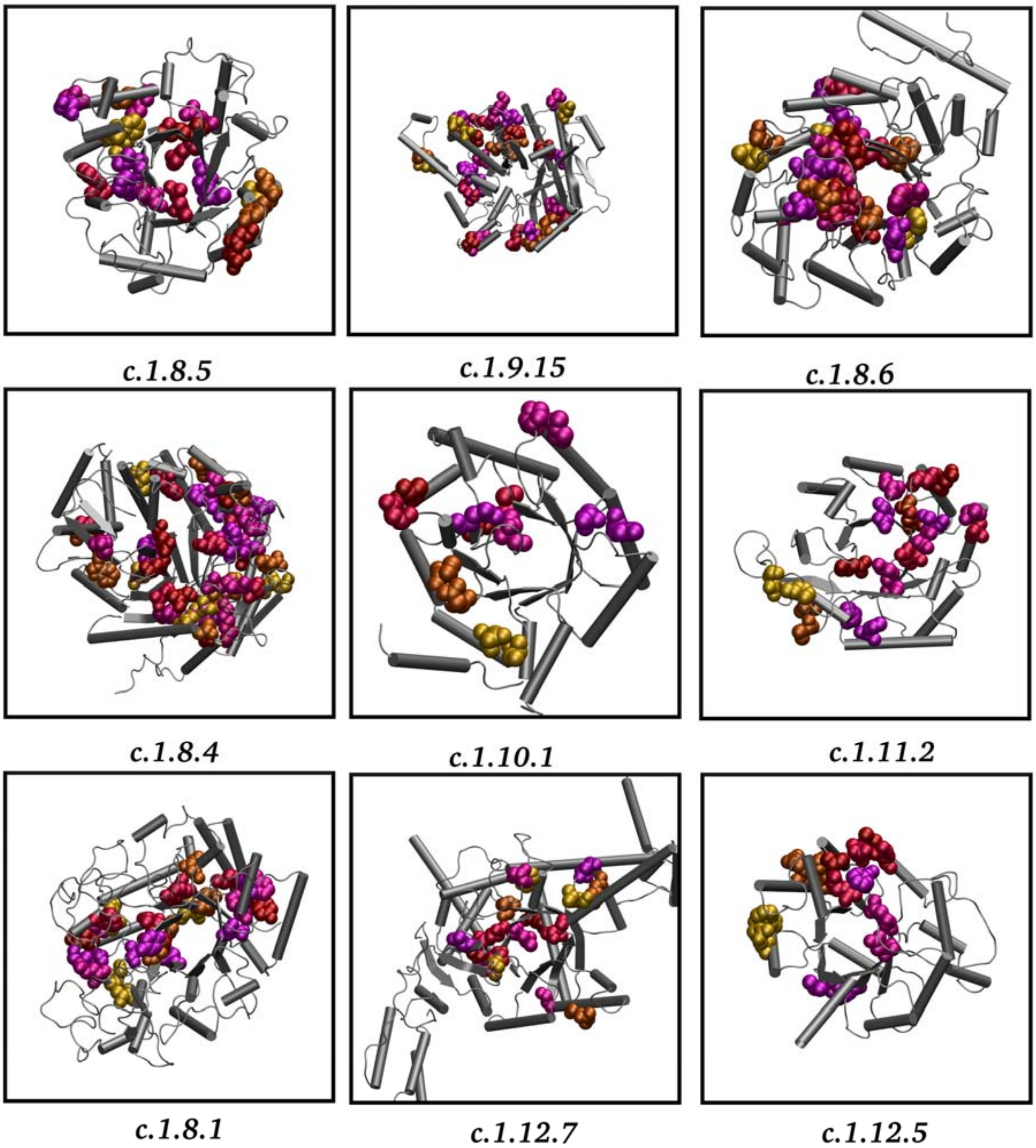

Figure S3 - Presence of conserved high energy interactions at the catalytic face of the TIM fold

The residues participating in the conserved interactions (*f*-$PEN_{-20(0.8)}$) at the loop regions of different families of the TIM fold are highlighted in various shades of red.
