# Supplementary material for: Insights into the Fold Organization of TIM Barrel from Interaction Energy Based Structure Networks
Source: PLoS Comput Biol. 2012 May 17;8(5):e1002505. doi: 10.1371/journal.pcbi.1002505 (PMC3355060; doi:10.1371/journal.pcbi.1002505)
Supplement: Figure S4 — Role of conserved interaction in loops in the catalysis of TIM fold. The above figure shows the conserved high–energy interactions involving loops (f–PEN–20(0.8)) in different families. The ligands are represented in vdW spheres colored according to their atom types while the residues involved in the conserved interactions are highlighted in different shades of red. (PDF) [file pcbi.1002505.s004.pdf]

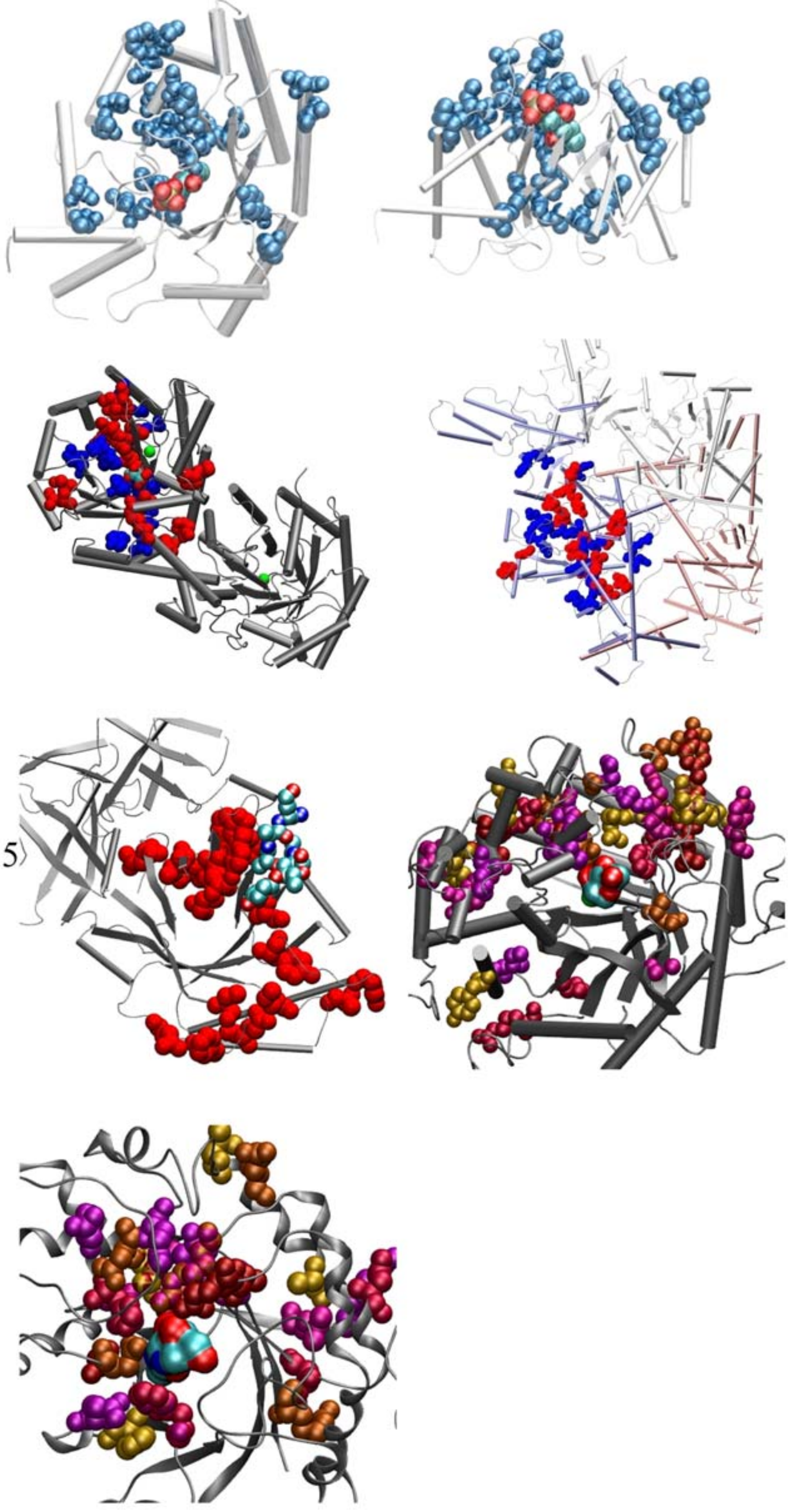

Figure S4 – Role of conserved interaction in loops in the catalysis of TIM fold.

The above figure shows the conserved high energy interactions involving loops (*f*-PEN$_{-20(0.8)}$) in different families. The ligands are represented in vdW spheres colored according to their atom types while the residues involved in the conserved interactions are highlighted in different shades of red. The blue spheres represent low energy

interactions (*f*-ljPEN$_{-7(0.8)}$). The interactions in c.1.12.7 is in and around the oligomerization regions thus probably helping them in the formation of higher order multimers.
