# Supplementary material for: Insights into the Fold Organization of TIM Barrel from Interaction Energy Based Structure Networks
Source: PLoS Comput Biol. 2012 May 17;8(5):e1002505. doi: 10.1371/journal.pcbi.1002505 (PMC3355060; doi:10.1371/journal.pcbi.1002505)
Supplement: Figure S5 — Family specific clusters from the f– PENs for selected families of the TIM fold. Clusters obtained from f–PENs are highlighted as spheres in different families of the TIM fold. High–energy clusters involving charged interactions at the core of the core β barrel residues that are obtained from the f–PENs–25(0.8) are distinguished by different shades of red. Low–energy vdW clusters that are obtained from f–ljPEN–8(1.0) are highlighted in different shades of blue in different families of the TIM fold. (PDF) [file pcbi.1002505.s005.pdf]

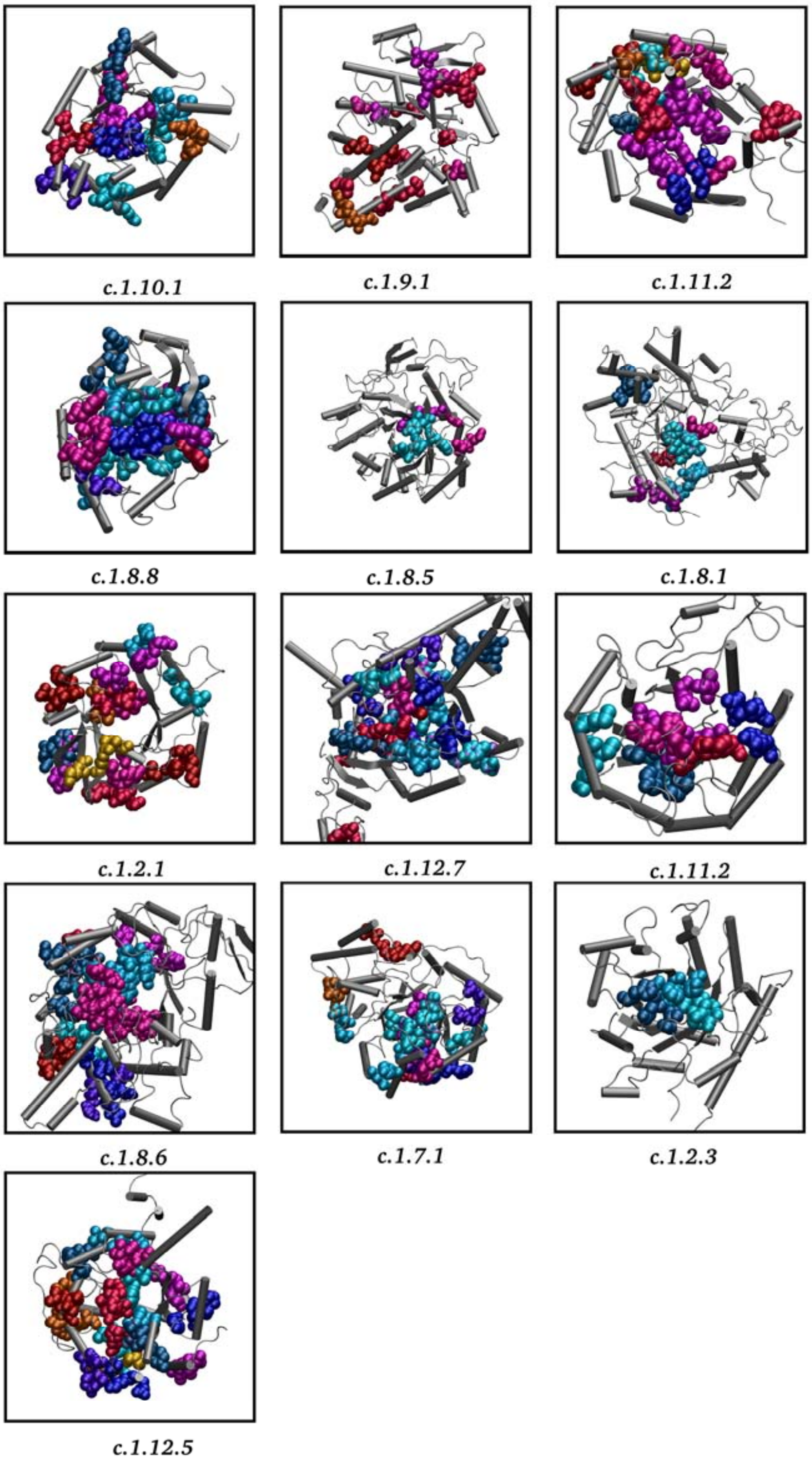

Figure S5 - Family specific clusters from the *f*-PENs for selected families of the TIM fold.

Clusters obtained from *f*-PENs are highlighted as spheres in different families of the TIM fold. High energy clusters involving charged interactions at the core of the core β barrel residues that are obtained from the *f*-PENs-25(0.8) are distinguished by different shades of red. Low energy vdW clusters that are obtained from *f*-ljPEN-8(1.0) are highlighted in different shades of blue in different families of the TIM fold.
