# Supplementary material for: Insights into the Fold Organization of TIM Barrel from Interaction Energy Based Structure Networks
Source: PLoS Comput Biol. 2012 May 17;8(5):e1002505. doi: 10.1371/journal.pcbi.1002505 (PMC3355060; doi:10.1371/journal.pcbi.1002505)
Supplement: Figure S6 — Conserved hubs present in some of the families of the TIM Barrel. The above figure shows the conserved hubs present in D–glucarate dehydratase–like (c.1.11.2, DGDL), Amylase (c.1.8.1, ACD), Aldo–keto reductases (c.1.7.1, AKR), HMGL–like (c.1.10.5, HMGL) and beta–N–acetylhexosaminidase (c.1.8.1, BNAH) families of the TIM Barrel domain. The hubs that are in shades of blue are from f–PEN–15(0.7) and that highlighted in shades of red are from f–PEN–7(1.0). (PDF) [file pcbi.1002505.s006.pdf]

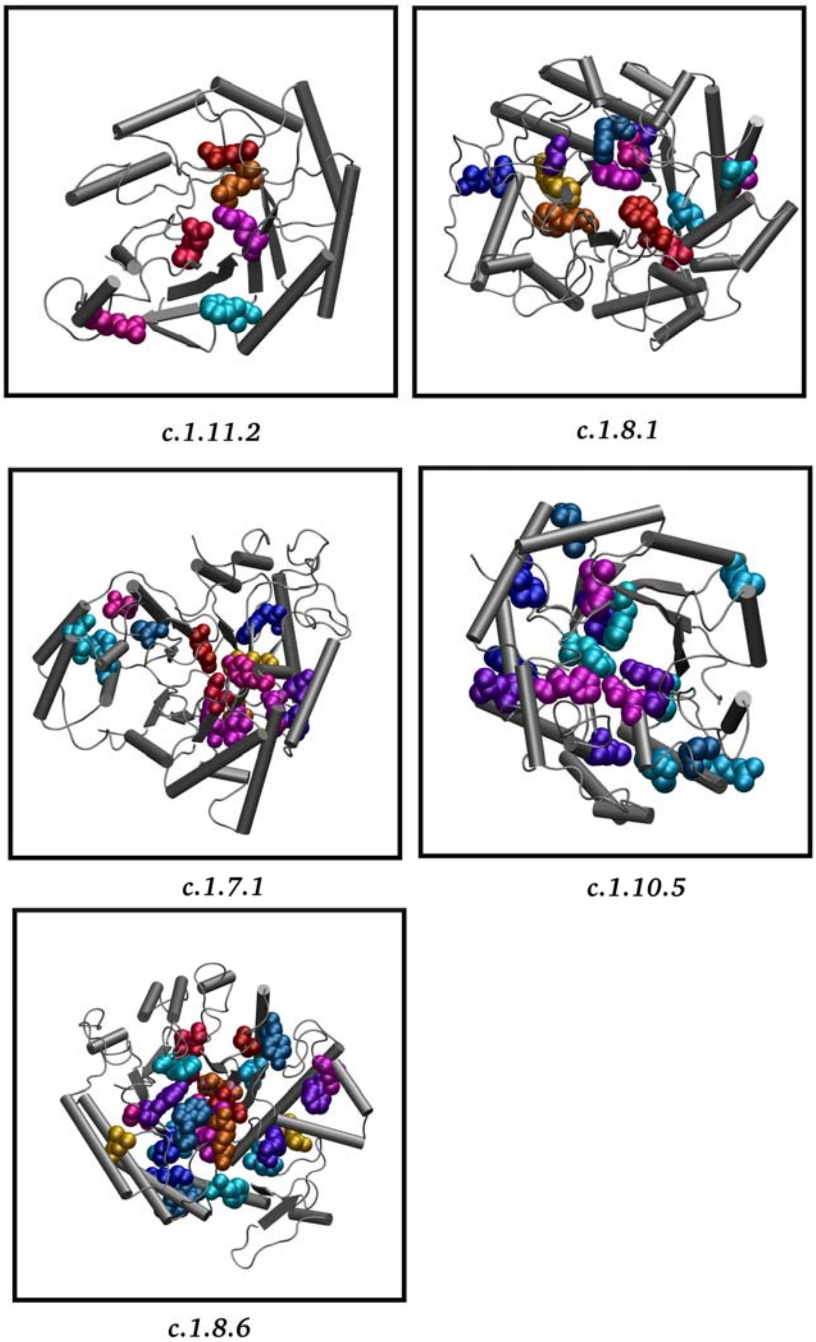

Figure S6 - Conserved hubs present in some of the families of the TIM Barrel.

The above figure shows the conserved hubs present in D-glucarate dehydratase-like (c.1.11.2, DGDL), Amylase (c.1.8.1, ACD), Aldo-keto reductases (c.1.7.1, AKR), HMGL-like (c.1.10.5, HMGL) and beta-N-acetylhexosaminidase (c.1.8.1, BNAH) families of the TIM Barrel domain. The hubs that are in shades of blue are from *f*-PEN-15(0.7) and that highlighted in shades of red are from *f*-PEN-7(1.0).
