# Supplementary material for: Insights into the Fold Organization of TIM Barrel from Interaction Energy Based Structure Networks
Source: PLoS Comput Biol. 2012 May 17;8(5):e1002505. doi: 10.1371/journal.pcbi.1002505 (PMC3355060; doi:10.1371/journal.pcbi.1002505)
Supplement: Figure S7 — Comparison between Cα distances between residues and the corresponding interaction energies. The above scatterplot shows the distances between the Cα atoms of residues plotted as a function of their interaction energies. It can be seen that although the interaction energy decreases as the cartesian distances between the Cα atoms decreases (green arrow), a number residue pairs fail to follow this behavior (red arrow, blue arrow and the points above the green arrow). This behavior can be attributed to high energy electrostatic interactions, cation-pi interactions, pi-pi interactions etc. that behave differently from contact based vdW interactions. Therefore, although most of the topology-based networks behave very well in studying many biophysical characteristics of proteins, we believe that PENs are capable of capturing the variations in the protein structures brought about by non-vdW interactions. In other words, while contact based networks are good at representing the width of the well that describes the interactions among protein residues, the energy based networks are capable of representing the depth of the well. (It should be noted that any Cα- Cα distance greater than 20 Å is considered as 20 Å and any Eij less than −30 kJ/mol is considered as −30 kJ/mol). (PDF) [file pcbi.1002505.s007.pdf]

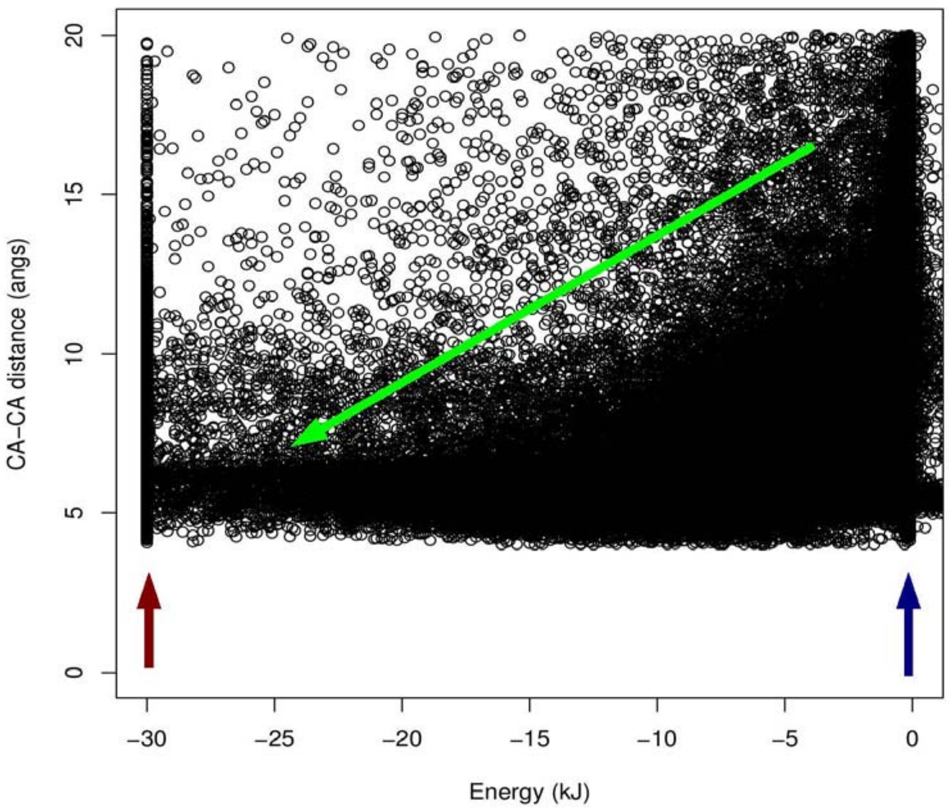

Figure S7 – Comparison between Cα distances between residues and the corresponding interaction energies.

The above scatterplot shows the distances between the Cα atoms of residues plotted as a function of their interaction energies. It can be seen that although the interaction energy decreases as the cartesian distances between the Cα atoms decreases (green arrow), a number residue pairs fail to follow this behavior (red arrow, blue arrow and the points above the green arrow). This behavior can be attributed to high energy electrostatic interactions, cation-pi interactions, pi-pi interactions etc. that behave differently from contact based vdW interactions. Therefore, although most of the topology-based networks behave very well in studying many biophysical characteristics of proteins, we believe that PENs are capable of capturing the variations in the protein structures brought about by non-vdW interactions. In other words, while contact based networks are good at representing the width of the well that describes the interactions among protein residues, the energy based networks are capable of representing the depth of the well. (It should be noted that any Cα- Cα distance greater than 20Å is considered as 20Å and any E_{ij} less than −30 kJ/mol is considered as −30 kJ/mol).
