# Supplementary material for: Insights into the Fold Organization of TIM Barrel from Interaction Energy Based Structure Networks
Source: PLoS Comput Biol. 2012 May 17;8(5):e1002505. doi: 10.1371/journal.pcbi.1002505 (PMC3355060; doi:10.1371/journal.pcbi.1002505)
Supplement: Table S1 — Different families of the TIM fold taken for analysis. (PDF) [file pcbi.1002505.s008.pdf]

## Table S1 Dataset of TIM fold

| S No | SCOP ID | Family Name | Abbreviations used | Total members |
|---|---|---|---|---|
| 1 | c.1.8.4 | Family 1 of glycosyl hydrolase | F1GH | 5 |
| 2 | c.1.8.6 | beta-N-acetylhexosaminidase catalytic domain | BNAH | 4 |
| 3 | c.1.8.3 | beta-glycanases | BG | 11 |
| 4 | c.1.10.1 | Class I aldolase | C1A | 3 |
| 5 | c.1.9.15 | PP1699/LP2961-like | PPL | 3 |
| 6 | c.1.2.1 | Histidine biosynthesis enzymes | HB | 3 |
| 7 | c.1.8.1 | Amylase, catalytic domain | ACD | 6 |
| 8 | c.1.10.5 | HMGL-like | HMGL | 2 |
| 9 | c.1.9.1 | Adenosine/AMP deaminase | ADA | 2 |
| 10 | c.1.11.2 | D-glucarate dehydratase-like | DGDL | 6 |
| 11 | c.1.2.4 | Tryptophan biosynthesis enzymes | WB | 3 |
| 12 | c.1.4.1 | FMN-linked oxidoreductases | FMNO | 6 |
| 13 | c.1.6.1 | Alanine racemase-like, N-terminal domain | ARLN | 3 |
| 14 | c.1.2.3 | Decarboxylase | DC | 5 |
| 15 | c.1.12.5 | HpcH/HpaI aldolase | HPXA | 3 |
| 16 | c.1.8.8 | 1,4-beta-N-acetylmuraminidase | BNAM | 3 |
| 17 | c.1.7.1 | Aldo-keto reductases (NADP) | AKR | 4 |
| 18 | c.1.12.7 | Phosphoenolpyruvate mutase/Isocitrate lyase-like | PEPM | 3 |
| 19 | c.1.8.5 | Type II chitinase | T2C | 4 |

*The families are aligned such that the top families have electrostatically dominated clusters in the central $\beta$- barrel. As one proceeds down the order the dominance of the electrostatics decrease and the dominance of vdW clusters increase.*
