# Supplementary material for: Insights into the Fold Organization of TIM Barrel from Interaction Energy Based Structure Networks
Source: PLoS Comput Biol. 2012 May 17;8(5):e1002505. doi: 10.1371/journal.pcbi.1002505 (PMC3355060; doi:10.1371/journal.pcbi.1002505)
Supplement: Table S2 — Dataset taken for SCA calculation. (PDF) [file pcbi.1002505.s009.pdf]

Table S2 Dataset taken for SCA calculation

Protein Name	Number of Sequences Considered	Ligand location
Xylanase	52	C-term
Endoglucanase CelA	23	-
Endoglucanase CelC	2	C-term
Beta-mannanase	11	-
Mannanase A	23	C-term
Plant beta-glucanases	2	-
beta-Galactosidase	34	C-term
beta-Glucuronidase	4	C-term
Xylanase A	49	C-term
Glucosylceramidase	4	C-term
Alpha-L-arabinofuranosidase	7	-
Beta-D-xylosidase	2	-
endo-1,4-beta-mannosidase	7	-
Endoglucanase H	102	C-term

All the protein sequences belonging to the β-glycanase family (according to SCOP) was culled for sequences with atleast less than 50% sequence identity and length between 200 and 500 residues. This yields 324 sequences from 15 proteins. The Multiple Sequence Alignment (MSA) was done using ClustalW. Alignment positions with less than 20% gaps we considered for SCA calculation. Default SCA parameters are used and the pairwise positional correlation varies from 0.2 – 0.6 majorly. The SCA code was obtained from Rama Ranganathan's Lab through personal communication. All the SCA results are mapped back to the structure of 1EDG for ease of structural mapping and comparison with structure based analyses (Figure 7).
