# Supplementary material for: Insights into the Fold Organization of TIM Barrel from Interaction Energy Based Structure Networks
Source: PLoS Comput Biol. 2012 May 17;8(5):e1002505. doi: 10.1371/journal.pcbi.1002505 (PMC3355060; doi:10.1371/journal.pcbi.1002505)
Supplement: Table S3 — Comparison of the “cluster residues” from f -PEN and the “sector residues” from SCA in β-glycanase family. (PDF) [file pcbi.1002505.s010.pdf]

Table S3: Comparison of the “cluster residues” from *f*-PEN and the “sector residues” from SCA in β-glycanase family

Cluster residues (*f*-PEN)*	Sector residues (SCA)*	Common residues (sectors ∩ clusters)*
72, 69, 65, 77, 117, 163, 118, 218, 164, 119, 248, 219, 165, 120, 166, 167, 220, 250, 302, 335, 303, 251, 304, 253, 337, 306, 338, 305, 336, 32, 170, 254, 307, 255, 308, 339, 34, 35, 169, 79, 33, 78, 116, 76, 115, 110, 106, 103, 107, 104, 111, 112, 108, 105, 109, 113, 71, 68, 64, 67, 63, 66, 73, 70, 75, 31, 141, 147, 146, 150, 148, 144, 142, 139, 145, 143, 140, 137, 197, 210, 207, 201, 204, 208, 209, 205, 199, 202, 206, 203, 200, 198, 196, 320, 329, 330, 326, 323, 324, 327, 328, 325, 322, 319	55, 73, 74, 81, 82, 83, 104, 106, 109, 110, 112, 114, 119, 123, 130, 158, 159, 160, 161, 163, 164, 165, 167, 169, 171, 199, 203, 206, 207, 219, 226, 228, 229, 230, 231, 237, 252, 255, 270, 272, 273, 277, 288, 290, 293, 300, 302, 318, 319, 321, 323, 328	163<br>164<br>119<br>219<br>165<br>167<br>302<br>255<br>169<br>110<br>106<br>104<br>112<br>109<br>73<br>207<br>199<br>206<br>203<br>323

** Residue numbers corresponding to PDB id 1EDG are given.*
